# Unlocking the Potential of the CA2, CA7, and ITM2C Gene Signatures for the Early Detection of Colorectal Cancer: A Comprehensive Analysis of RNA-Seq Data by Utilizing Machine Learning Algorithms

**DOI:** 10.3390/genes14101836

**Published:** 2023-09-22

**Authors:** Neha Shree Maurya, Sandeep Kushwaha, Ramesh Raju Vetukuri, Ashutosh Mani

**Affiliations:** 1 Department of Biotechnology, Motilal Nehru National Institute of Technology Allahabad, Prayagraj 211004, India; nmaurya@mnnit.ac.in; 2 National Institute of Animal Biotechnology, Hyderabad 500032, India; sandeep@niab.org.in; 3 Department of Plant Breeding, Swedish University of Agricultural Sciences, 23053 Alnarp, Sweden

**Keywords:** colorectal cancer, feature selection, machine learning, gene expression, gene signatures, correlation

## Abstract

Colorectal cancer affects the colon or rectum and is a common global health issue, with 1.1 million new cases occurring yearly. The study aimed to identify gene signatures for the early detection of CRC using machine learning (ML) algorithms utilizing gene expression data. The TCGA-CRC and GSE50760 datasets were pre-processed and subjected to feature selection using the LASSO method in combination with five ML algorithms: Adaboost, Random Forest (RF), Logistic Regression (LR), Gaussian Naive Bayes (GNB), and Support Vector Machine (SVM). The important features were further analyzed for gene expression, correlation, and survival analyses. Validation of the external dataset GSE142279 was also performed. The RF model had the best classification accuracy for both datasets. A feature selection process resulted in the identification of 12 candidate genes, which were subsequently reduced to 3 (CA2, CA7, and ITM2C) through gene expression and correlation analyses. These three genes achieved 100% accuracy in an external dataset. The AUC values for these genes were 99.24%, 100%, and 99.5%, respectively. The survival analysis showed a significant logrank *p*-value of 0.044 for the final gene signatures. The analysis of tumor immunocyte infiltration showed a weak correlation with the expression of the gene signatures. CA2, CA7, and ITM2C can serve as gene signatures for the early detection of CRC and may provide valuable information for prognostic and therapeutic decision making. Further research is needed to fully understand the potential of these genes in the context of CRC.

## 1. Introduction

Colorectal cancer (CRC) is a type of cancer that affects the colon or rectum, which are parts of the large intestine. CRC is a prevalent form of cancer globally, with an annual occurrence of 1.1 million new cases. It also holds the distinction of being the second-largest contributor to cancer-related mortality [1]. Recent studies have documented a significant increase in the incidence of CRC among individuals below the age of 50 [2]. Projections indicate that the incidence of CRC will escalate to 3.2 million new cases and 1.6 million deaths by 2040. The vast majority of these cases are anticipated to be concentrated in countries with a high Human Development Index (HDI) [3].

The treatment of CRC imposes a heavy burden on both patients and society. Most CRC cases are sporadic and arise from colorectal adenocarcinoma (CRA), 80% of which possess APC mutations [4]. These mutations result in multiple genetic alterations, including p53, KRAS, and SMAD4 mutations, ultimately leading to malignant transformations [5]. The availability of CRC gene expression data has grown exponentially over time and enabled researchers to analyze these data utilizing multiple Machine Learning (ML) approaches to find suitable diagnostic biomarkers and gene signatures for CRC [6].

A recent study by Wang et al. identified the core genes that have roles in the development and oncogenesis of CRA by utilizing CRC datasets from the gene expression omnibus (GEO). A variety of analytical techniques were utilized in this study, including functional pathway enrichment, a protein–protein interaction network analysis, a stem correlation analysis, a CIBERSORT analysis, and a survival analysis. The findings were further validated through RT-qPCR and immunohistochemical staining [7]. Fu et al. conducted a study to develop a prognostic model for stomach adenocarcinoma (STAD) based on tumor mutation burden (TMB). They utilized gene expression data from the TCGA and GEO databases to identify a set of immune markers responsible for STAD progression. The resulting immune prognostic model was then used to create a dynamic nomograph application for clinical use [8]. Su et al. developed an ML model for classifying colon cancer samples based on gene expression information. These markers can diagnose the stage of cancer in time and help in improving its prognosis with timely treatment. The RF model provided the best results for the diagnosis of colon cancer, with an average accuracy of 99.81%. The study also identified eight genes, including GCNT2, GLDN, SULT1B1, UGT2B15, PTGDR2, GPR15, BMP5, and CPT2, which were found to be associated with colon cancer prognosis [9].

This study aims to discover significant gene signatures for CRC using transcriptomic profiling data from the TCGA and GEO datasets. Machine learning algorithms combined with feature selection techniques were applied to distinguish between tumor and normal CRC samples. The significance of the identified genes was evaluated by examining their biological functions and pathways in the context of CRC sample classification.

## 2. Materials and Methods

### 2.1. CRC Gene Expression Dataset Collection

The TCGA-CRC mRNA expression dataset was downloaded from NIH-GDC (Genomic Data Commons DataPortal) https://portal.gdc.cancer.gov-/ (accessed on 2 March 2023) through Bioconductor R package TCGAbiolinks [10]. The mRNA dataset contained 695 samples, including 644 CRC tissue samples and 51 normal tissue samples. The GEO dataset GSE50760 [11,12,13] for CRC gene expression data was downloaded from https://www.ncbi.nlm.nih.gov/geo/ (accessed on 20 March 2023). The GEO dataset had 54 samples, where 18 were normal tissue samples, and the remaining 36 were tumor tissue samples. All the CRC samples of the current study used to understand and differentiate the expression patterns between normal and tumor tissues involved samples from normal colon, primary, and metastasized CRC, ranging from cancer stage I to IV.

### 2.2. Feature Selection on CRC Gene Expression Datasets

The CRC datasets were initially balanced using a sample resampling technique. The resampling technique used in our study was the Synthetic Minority Oversampling Technique (SMOTE), which synthesizes new minority data between existing minority data. It randomly picks up the minority class and calculates the K-nearest neighbor for that particular point. Since unbalanced datasets tend to provide biased classification results for sample classes, this resampling technique helps to reduce the bias of machine learning algorithms when performing classification against the major class. The feature selection method LASSO helps to select the best set of features, which helps in providing a better classification accuracy.

The Least Absolute Shrinkage and Selection Operator (LASSO), a feature selection algorithm, was used to narrow down the number of genes, as the high dimensionality of the dataset makes it difficult to classify the data. The LASSO algorithm constructed a linear model and penalized the regression coefficients with L1 distance. Most coefficients were reduced to zero, and the remaining inputs were selected.

The ML algorithms adaboost [14], random forest (RF) [15], linear regression (LR) [16], gaussian naive bayes (GNB) [17], and support vector machine (SVM) [18,19,20,21] were implemented on the features obtained from the LASSO. The model accuracies were analyzed for both CRC datasets. The common features obtained after applying the LASSO on both CRC datasets were selected for further analysis.

### 2.3. Cumulative Gene Expression and Correlation Analysis of the Selected Features

The Gene Expression Profiling Interactive Analysis (GEPIA2) online database (http://gepia.cancer-pku.cn/) (accessed on 20 March 2023). [22] was used for a cumulative expression analysis of the selected features by using parameters matching the TCGA normal data with logFC > |1.5| and a *p*-value cutoff of 0.01. Multiple gene comparison was also performed for the selected features through GEPIA2, where they were matched with normal TCGA data. The gene expression of the selected common genes for both CRC datasets was analyzed for the count data information.

A correlation analysis was also performed for both CRC datasets utilizing the R package “GGally”, and genes that had correlation coefficient values of more than 0.5 in both datasets were selected for further analysis.

### 2.4. External CRC Dataset Validation and ROC Curve Analysis

The external CRC dataset GSE142279 was used to validate the identified features of importance by performing classification using an RF-based classification model, and then the dataset was cross-validated through an ROC curve analysis to check their diagnostic efficiency. An overall survival analysis was also performed for these significant features of importance through the GEPIA server.

### 2.5. Analysis of Association of Final Set of Gene Expression with Tumor-Infiltrating Immunocytes

The TISIDB web portal (http://cis.hku.hk/TISIDB/) (accessed on 20 March 2023). [23] was utilized to explore the relationship between the expressions of the finalized genes in our study and tumor-associated infiltrating immunocytes, including CD4+ T-cells, CD8+ T-cells, dendritic cells, macrophages, B-cells, and neutrophils. TISIDB helped to analyze the clinical and transcriptomics data across 30 cancer types from the TCGA database.

### 2.6. Biological Function, Literature, and Pathway Analysis of the Selected Gene Signatures

A biological, functional, and literature analysis was performed to validate the gene signatures obtained from the procedure mentioned above. The pathway associated with each gene was studied using string Db [24] and the Cytoscape tool.

## 3. Results

### 3.1. Dataset Overview and Its Preprocessing

**Figure 1:**
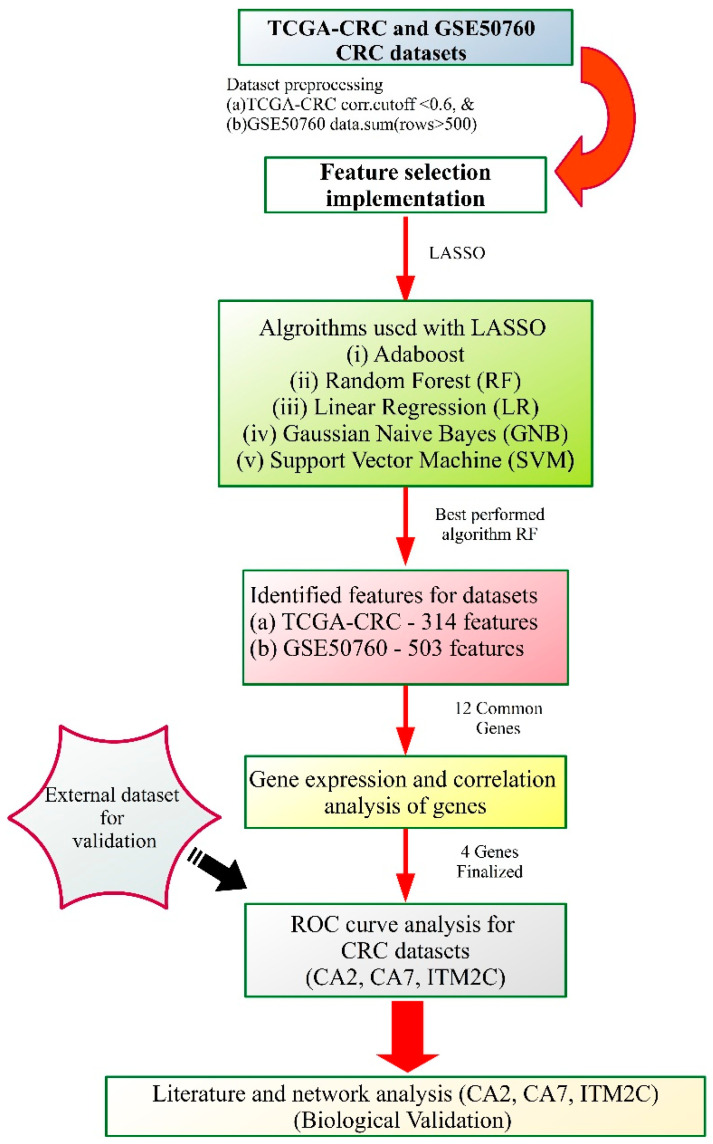
Workflow for the identification of gene signatures for colorectal cancer through feature selection approach with different machine learning algorithms. TCGA: The Cancer Genome Atlas; CRC: Colorectal cancer; LASSO: Least Absolute Shrinkage and Selection Operator; RF: Random Forest; LR: Linear Regression; GNB: Gaussian Naive Bayes; SVM: Support Vector Machine; and ROC: Receiver operating characteristics.

**Figure 2:**
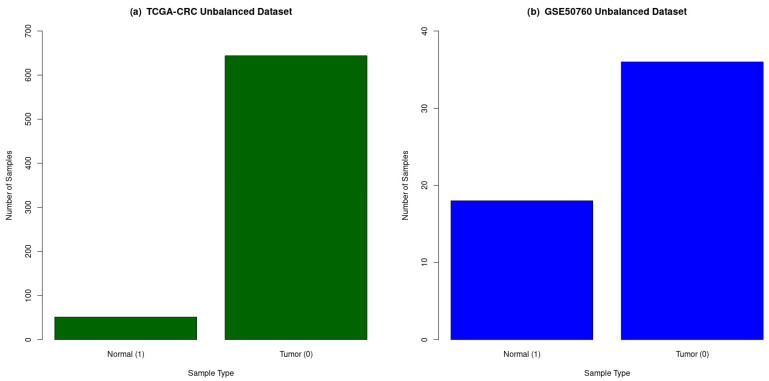
The distribution of normal and tumor samples in original and unbalanced CRC datasets. (**a**) TCGA-CRC had 51 normal tissue samples and 644 tumor tissue samples, and (**b**) GSE50760 had 18 normal tissue samples and 36 tumor tissue samples. Green: TCGA-CRC unbalanced dataset; and Blue: GSE50760 unbalanced dataset.

A total of 695 and 54 CRC samples were collected from the TCGA and GEO repositories, respectively, and are described in Table 1, while a workflow of the study is shown in Figure 1. The distribution of the CRC samples in each dataset based on the tumor and normal classes shown in Figure 2. The normalized TCGA-CRC mRNA data were obtained using the following parameters: data type—Gene Expression Quantification, workflow type—HTSeq-Counts, and a correlation cut-off of 0.6 for genes. The TCGA-CRC dataset comprised 23,187 features, while the GSE50760 dataset initially had 35,238 features. It was filtered for low gene counts using the criteria of “data.sum(axis = 0) > 500”, resulting in 17,300 features. 

### 3.2. Feature Selection and Machine Learning Model Implementation

**Figure 3:**
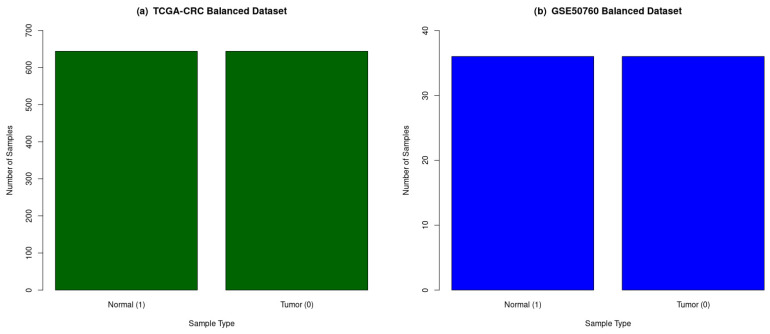
The distribution of normal and tumor samples in balanced CRC datasets. (**a**) TCGA-CRC has 644 normal tissue samples and 644 tumor tissue samples, and (**b**) GSE50760 has 36 normal tissue samples and 36 tumor tissue samples. Green: TCGA-CRC balanced dataset; and Blue: GSE50760 balanced dataset.

The CRC datasets’ (TCGA-CRC and GSE50760) gene expression dataset was balanced using a resampling technique before implementing the LASSO, as shown in Figure 3, because unbalanced datasets tend to provide biased classification results for sample classes. The feature selection method LASSO helped to select the best set of features, which helped in providing a better classification accuracy.

**Figure 4:**
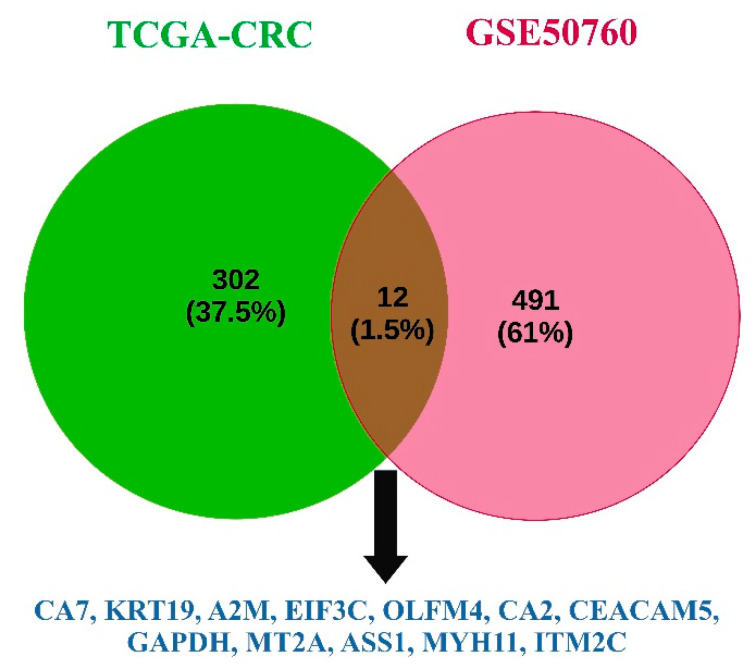
List of 12 common genes (CA7, KRT19, A2M, EIF3C, OLFM4, CA2, CEACAM5, GAPDH, MT2A, ASS1, MYH11, and ITM2C) identified after feature selection technique LASSO analysis from TCGA-CRC and GSE50760 CRC datasets.

For the CGA-CRC and GSE50760 datasets, the LASSO provided 314 and 503 features, respectively. These sets of features were utilized by different ML models (adaboost, RF, LR, GNB, and SVM) to analyze the classification accuracy, as described in Table 2. The RF gave maximum classification accuracies of 100% with the TCGA-CRC dataset and 94.44% with the GSE50760 dataset. A total of 12 common genes, namely CA7, KRT19, A2M, EIF3C, OLFM4, CA2, CEACAM5, GAPDH, MT2A, ASS1, MYH11, and ITM2C, were found from both CRC datasets, as shown in Figure 4.

### 3.3. Cumulative Gene Expression and Correlation Analysis of the Selected Features

**Figure 5:**
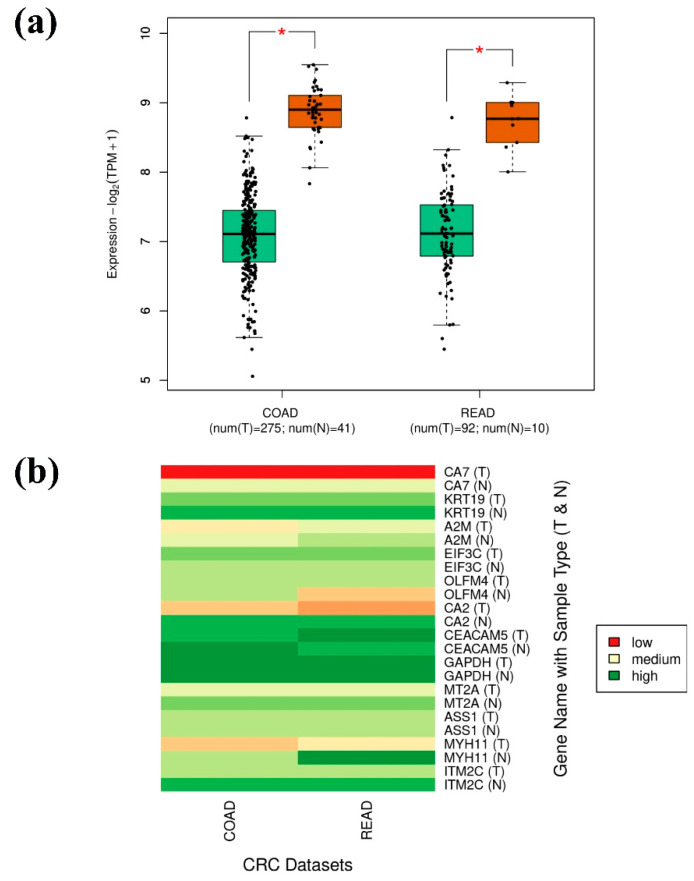
Cumulative gene expression analysis of 12 genes (CA7, KRT19, A2M, EIF3C, OLFM4, CA2, CEACAM5, GAPDH, MT2A, ASS1, MYH11, and ITM2C) from TCGA-CRC dataset through GEPIA2 server. (**a**) Box-plot for TCGA-CRC dataset between tumor (Green) and normal (Brown) samples with the asterisk mark showing statistical significance, and (**b**) comparative gene expression heatmap between tumor and normal samples (Green: high expression, cream: medium expression, and red: low expression).

A cumulative gene expression analysis for the final 12 genes showed a significant downregulation when compared to the normal TCGA-CRC samples, as shown in Figure 5a. A multiple gene comparison analysis also proved the downregulation of the final obtained 12 genes through a heatmap shown in Figure 5b. It is evident through the heatmap that maximum downregulation was observed with CA7 and minimum downregulation was observed with GAPDH when compared to the TCGA-CRC (tumor and normal) samples.

**Figure 6:**
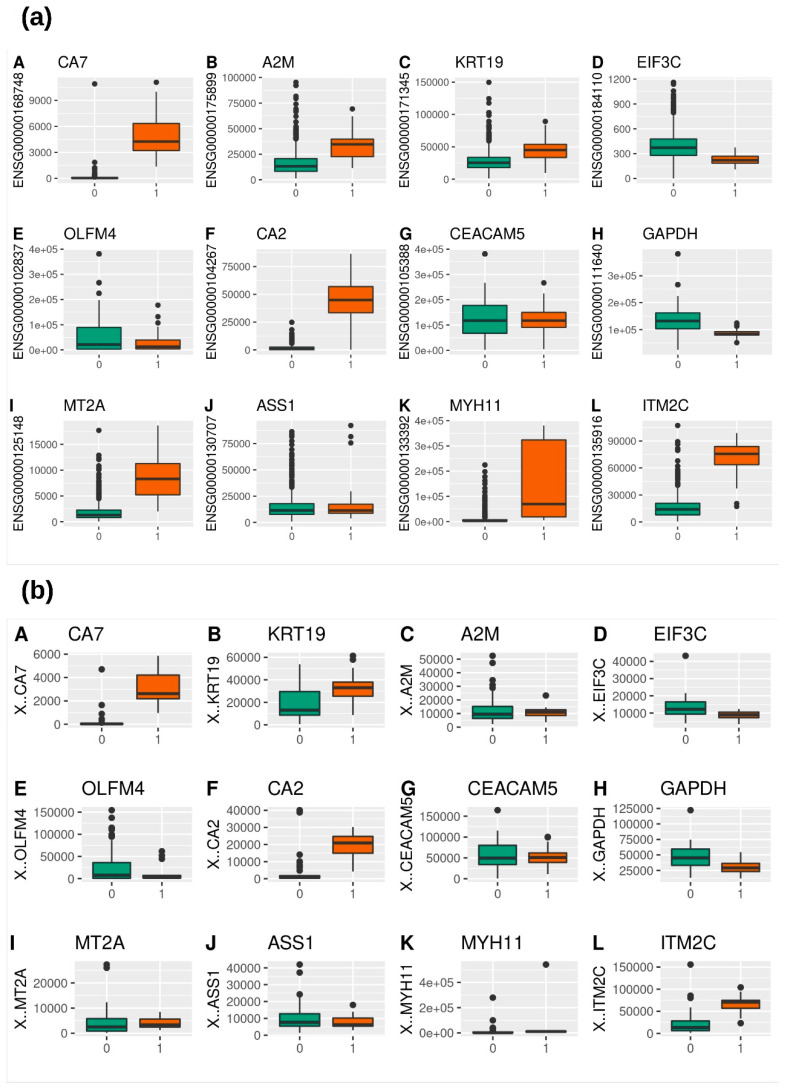
Gene expression analysis for CRC datasets of 12 shortlisted genes CA7, KRT19, A2M, EIF3C, OLFM4, CA2, CEACAM5, GAPDH, MT2A, ASS1, MYH11, and ITM2C. (**a**) TCGA-CRC dataset, and (**b**) GSE50760 dataset. Green color denotes tumor (0 class code) samples and brown color denotes normal (1 class code) samples.

The gene expression analysis for the TCGA-CRC and GSE50760 datasets shown in Figure 6 also proved a similar downregulation trend to that observed through the GEPIA2 server. 

**Figure 7:**
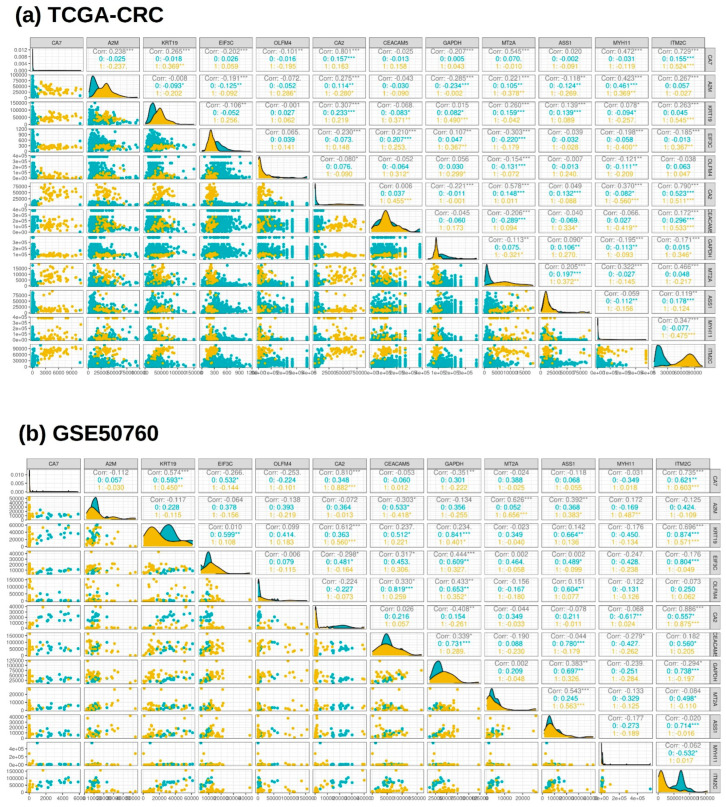
Correlation analysis between 12 common genes (CA7, KRT19, A2M, EIF3C, OLFM4, CA2, CEACAM5, GAPDH, MT2A, ASS1, MYH11, and ITM2C) identified through LASSO. (**a**) TCGA-CRC dataset, and (**b**) GSE50760 dataset. Yellow color denotes normal sample class with code 1, while blue color denotes tumor sample class with code 0.

The correlation analysis for the final set of 12 genes showed a strong correlation for the genes that were significantly downregulated when compared to the normal samples for both CRC datasets, as shown in Figure 7. A list of three genes (CA7, CA2, and ITM2C) were shortlisted based on the gene expression and correlation analyses. The TCGA-CRC dataset showed a significant positive correlation value between CA7 and CA2, which was 0.8, while CA7–ITM2C was 0.72 and CA2−ITM2C was 0.79. A similar positive correlation was observed for the CRC dataset GSE50760, where the correlation value between CA7 and CA2 was 0.8, while CA7−ITM2C was 0.73 and CA2−ITM2C was 0.88.

### 3.4. External CRC Dataset Validation and ROC Curve Analysis

**Figure 8:**
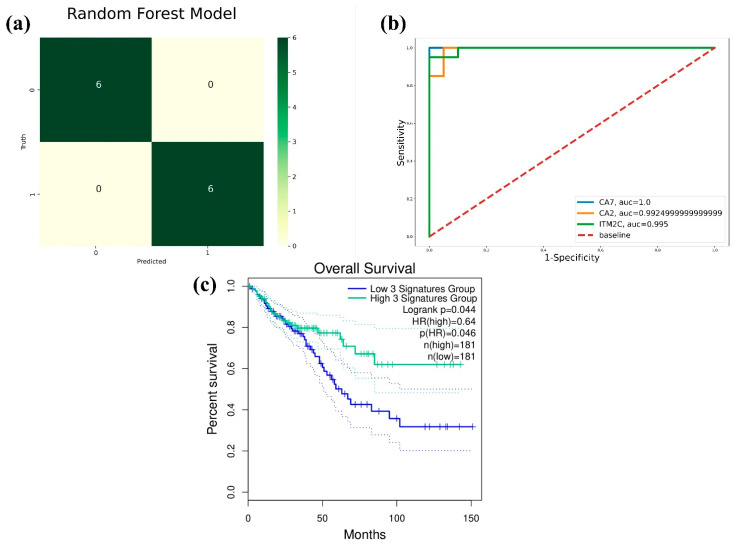
The external dataset (GSE142279) performance validation of RF-based ML model for CRC classification with CA7, CA2, and ITM2C gene expression count data. (**a**) Confusion matrix between truth and predicted classes, where 0 is for tumor sample and 1 is for normal sample, (**b**) ROC curve analysis with AUC > 0.99, and (**c**) overall survival analysis with logrank *p* = 0.044 for gene signatures.

The external CRC dataset GSE142279 (40 CRC sample size) was classified utilizing three significant gene expression count data with an RF-based ML model and provided a classification accuracy of 100%. The performance metrics of the validation dataset are described in Table 3, which clearly shows the performance of the ML model. The confusion matrix shown in Figure 8a displays the distribution of six samples in each class, i.e., the tumor and normal classes. The ROC curve analysis indicated that the AUC values for the CA7, CA2, and ITM2C genes were 1.0, 0.992, and 0.995, respectively, as shown in Figure 8b. The overall survival analysis plot, obtained through the GEPIA2 server, showed a statistically significant logrank *p*-value of 0.044 for all three gene signatures (CA7, CA2, and ITM2C), as shown in Figure 8c.

### 3.5. Analysis of Association of Final Set of Gene Expression with Tumor Infiltrating Immunocytes

**Figure 9:**
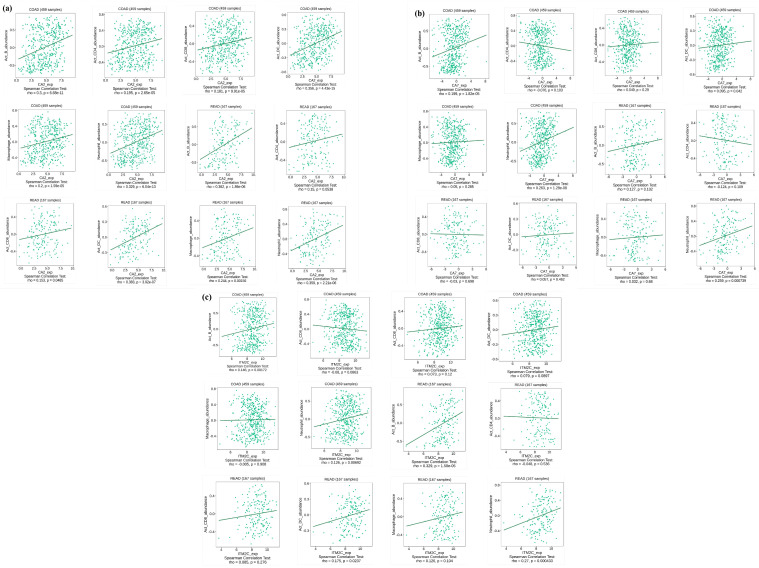
Relationship of gene expression of CA2, CA7, and ITM2C with immunocyte infiltration, including CD4+ T-cells, CD8+ T-cells, dendritic cells, macrophages, B-cells, and neutrophils in CRC. (**a**) CA2, (**b**) CA7, and (**c**) ITM2C.

The TISIDB web server was utilized for exploring the relationship between the gene signatures’ (CA7, CA2, and ITM2C) expression levels and the immunocyte infiltration level in the CRC samples. The gene expressions of CA2, CA7, and ITM2C showed a weak correlation with immunocyte infiltration, as shown in Figure 9. CA2 expression exhibited a strong correlation with the Act_B (rho = 0.3, *p* = 6.68 × 10^−11^), Act_DC (rho = 0.356, *p* = 4.43 × 10^−15^), and Neutrophils (0.329, *p* = 6.04 × 10^−13^) levels in colon cancer and with the Act_B (rho = 0.362, *p* = 1.86 × 10^−6^), Act_DC (rho = 0.38, *p* = 3.92 × 10^−7^), and Neutrophils (rho = 0.359, *p* = 2.21 × 10^−6^) levels in rectal cancer. CA7 expression exhibited a strong correlation with the Act_B (rho = 0.199, *p* = 1.82 × 10^−5^) and Neutrophils (0.263, *p* = 1.28 × 10^−8^) levels in colon cancer and with the Act_B (rho = 0.127, *p* = 0.102) and Neutrophils (rho = 0.259, *p* = 0.000739) levels in rectal cancer. ITM2C expression exhibited a strong correlation with the Act_B (rho = 0.146, *p* = 0.00172) and Neutrophils (rho = 0.126, *p* = 0.00692) levels in colon cancer and with the Act_B (rho = 0.329, *p* = 1.58 × 10^−5^) and Neutrophils (rho = 0.27, *p* = 0.000433) levels in rectal cancer. 

### 3.6. Biological Function and Pathway Analysis

The biological functions of the final gene signatures of CA2, CA7, and ITM2C were studied and are described in Table 4. Carbonic anhydrase 2 and Carbonic anhydrase 7 (CA2 and CA7) belong to the CA family isozymes, which catalyze the reversible hydration of carbon dioxide. CA2 is involved in many critical physiological or biochemical processes based on ion transport and pH balance, such as respiration, digestion, bone resorption, and renal acidification [25]. The CA2 Gene Ontology (GO) annotations related to this gene include arylesterase activity and carbonate dehydratase activity. CA7 shows extensive diversity in tissue distribution and in their subcellular localization. The cytosolic protein encoded by this gene is predominantly expressed in the brain and the colon and contributes to bicarbonate-driven GABAergic neuron excitation. The integral membrane protein 2C (ITM2C) is broadly expressed in the colon and brain. The Gene Ontology (GO) annotations related to this gene include amyloid β binding.

**Figure 10:**
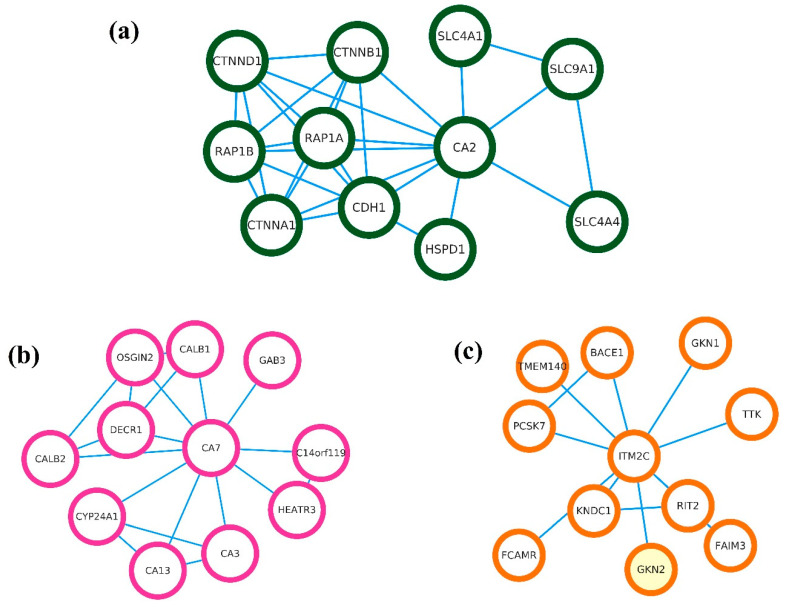
String network showing protein–protein interactions for final CRC gene signatures. (**a**) CA2, (**b**) CA7, and (**c**) ITM2C.

The string network for the CRC gene signatures is shown in Figure 10. The string network interactors for CA2 are SLC9A1, SLC4A4, SLC4A1, HSPD1, CDH1, CTNNB1, CTNND1, RAP1B, CTNNA1, and RAP1A. The disease gene association from the CA2 string network for colorectal cancer was found to be prominent with the CTNNB1 (Z-score—7.8) and CDH1 (Z-score—7.2) genes. The string network interactors of CA7 are CYP24A1, OSGIN2, DECR1, CALB2, C14orf119, GAB3, HEATR3, CALB1, CA3, and CA13. The string network interactors for ITM2C include TTK, PCSK7, FCAMR, RIT2, FAIM3, KNDC1, TMEM140, BACE1, GKN2, and GKN1.

## 4. Discussion

Advances in colorectal cancer diagnostic technology have ushered in new trends in the early detection of CRC. The most recent trends include virtual colonoscopy, improved stool-based tests, and genetic tests. Currently, ML algorithms are also increasingly being used to help in diagnosing CRC. In particular, they are used to classify normal and malignant tumorous tissues. In this study, we utilized gene expression count data from the TCGA-CRC and GSE50760 CRC datasets. The TCGA-CRC dataset was preprocessed with a corr.cutoff of <0.6 and GSE50760 with data.sum(rows > 500). 

The feature selection technique LASSO was implemented with five different ML algorithms, namely Adaboost, RF, LR, GNB, and SVM. The supervised ML algorithms used in the current study have certain limitations, such as the adaboost algorithm being sensitive towards noisy data and vulnerable to overfitting, RF sometimes being biased towards dominant classes in imbalanced datasets, LR being sensitive towards outliers and unable to capture complex patterns, GNB not being able to capture complex relationships between features, which might lead to its suboptimal performance, and SVM being computationally intensive in nature. Since every ML algorithm has certain limitations, to overcome these limitations, ML algorithms based on different working principles were selected.

The classification accuracies obtained after feature selection with the TCGA-CRC dataset were 99.94 (adaboost), 100 (RF), 99.93 (GNB), 100 (LR), and 100 (SVM), and with the GSE50760 dataset were 94.16 (adaboost), 94.44 (RF), 93.21 (GNB), 93.40 (LR), and 92.12 (SVM). Since the maximum accuracy with LASSO was obtained with the RF ML model in both the CRC datasets, features of importance were extracted.

The TCGA-CRC dataset had filtered 314 and GSE50760 had 503 features that were important, and the Venn diagram analysis found 12 common genes. The common genes, namely, were CA7, KRT19, A2M, EIF3C, OLFM4, CA2, CEACAM5, GAPDH, MT2A, ASS1, MYH11, and ITM2C. Gene expression and correlation analyses were performed for both the CRC datasets, and it was observed that the CA2, CA7, and ITM2C genes had shown a significant downregulation in the tumor samples when compared to the normal CRC samples, and they all had a high positive correlation in terms of gene expression.

To justify the results, the external CRC dataset was utilized to observe the gene expression patterns of the CA2, CA7, and ITM2C genes. The external dataset had 40 samples, in which there was a 1:1 sample distribution for the tumor and normal CRC samples. The dataset was divided into training and testing data in a 7:3 ratio and was classified based on the gene expression of the final gene signatures with the RF model. The dataset achieved a 100% classification accuracy, and the ROC analysis gave AUC values for the CA2, CA7, and ITM2C of 99.24%, 100%, and 99.5%, respectively. The overall survival analysis through the GEPIA2 server for the final CRC gene signatures had a statistically significant logrank *p*-value = 0.044. The tumor immunocyte infiltration analysis showed a weak correlation between the gene signature expression and immunocyte infiltration levels. The most positive correlation was observed with the CA2 gene expression for Act_DC (rho = 0.356, *p* = 4.43 × 10^−15^) in colon and Act_DC (rho = 0.38, *p* = 3.92 × 10^−7^) in rectal cancer.

CA2 is a member of the human Carbonic Anhydrases group of metal enzymes, which convert carbon dioxide into bicarbonate [7]. CA2 variants have been associated with various health conditions such as osteoporosis, cancer, ulcers, and obesity [26]. The reduced expression of CA2 may enhance the stem-cell-like characteristics of adenoma cells in CRC and could potentially serve as a marker for high-risk CRC adenomas. Recent investigations have revealed that CA2 exerts a significant inhibitory effect on the proliferation of CRC cells by inducing cell cycle arrest at both the G0/G1 and G2 phases in a well-established SW480 CRC cell line [27]. Studies have shown that patients with a high CA2 expression have better disease-free survival and overall survival than those with a low expression in hepatocellular carcinoma [28]. A low CA2 expression negatively correlates with pathological state, distant metastasis, and tumor size in gastric cancer [29,30]. 

The CA7 expression was downregulated in a microarray-based gene expression profiling study, where a prediction was made by ML models, namely a prediction analysis of microarray, artificial neural network, classification, and regression trees [31]. Another study identified the downregulation of CA7 in CRC, where it acts as a tumor suppressor gene. The reduced expression of CA7 in CRC is associated with positive lymph node metastasis and poor differentiation. The survival analysis results also revealed that patients with a higher expression of CA7 tended to have a longer disease-specific survival time, compared to those with a lower expression of CA7 [32].

ITM2C showed downregulation in the CRC datasets explored for the study. Other members of the ITM protein family, like ITM2A, have been studied, and it was found that it acts as a tumor suppressor gene in breast cancer. It was observed that high expression levels of ITM2A are associated with more prolonged overall survival and relapse-free survival in patients with colorectal cancer. Additionally, the overexpression of ITM2A has been found to inhibit tumor cell proliferation and reduce their capacity for invasion and migration [33]. Since no research has been reported for the role of ITM2C related to CRC progression, based on the above evidence, we conclude that ITM2C can act as a tumor suppressor gene, meaning that it can help in regulating cell growth and division.

The genes CA2, CA7, and ITM2C collectively play critical roles in CRC progression, with CA2 influencing adenoma characteristics and cell proliferation, CA7 acting as a tumor suppressor gene, and ITM2C likely sharing similar tumor-suppressive properties with its family member ITM2A. In summary, we determined that CA2, CA7, and ITM2C can be utilized as gene signatures for CRC. They can act as potential therapeutic targets for improved prognoses in CRC patients. However, our work has certain limitations. First, gene expression validation based on RT-PCR needs to be performed to justify our results. Second, more datasets need to be tested to validate the accuracy and significance of the developed ML model. Furthermore, basic research needs to be performed to verify our ML model and to understand the basic regulatory mechanism for these gene signatures in vitro and in vivo.

## 5. Conclusions

In conclusion, this study showed that the RF model provided the highest accuracy in classifying normal and malignant tumorous CRC tissues. The LASSO identified 12 common genes (CA2, CA7, and ITM2C, etc.) that were analyzed for their gene expression and correlation. Further analysis of an external CRC dataset and various literature sources revealed that CA2, CA7, and ITM2C could act as gene signatures for the early detection of CRC and showed the potential to regulate cell growth and division in a manner that could prevent cancer progression. However, further validation through RT-PCR and additional datasets are needed to strengthen the results and understand the underlying regulatory mechanisms.

## Figures and Tables

**Table 1:** Sample distribution for normal and tumor classes with feature information.

Sample Groups	Number of Normal Samples	Number of CRC Samples	Total Samples	Number of Genes (Features)
TCGA-CRC	51	644	695	23,187
GSE50760	18	36	54	35,238

**Table 2:** Accuracy of different ML algorithms after feature selection through LASSO.

Sample Groups	ML Algorithms Accuracy with LASSO (%)				
	Adaboost	Random Forest	Gaussian Naive Bayes	Support Vector Machine (SVM)	Linear Regression
TCGA-CRC	99.94	100	99.93	100	100
GSE50760	94.16	94.44	93.21	93.40	92.12

**Table 3:** Classification performance report of external validation dataset GSE142279 through RF-based ML model for CRC classification.

Sample Class	Number of CRC Samples	Precision (PPV)	Recall (Sensitivity)	f1-Score
0 (Tumor)	6	100%	100%	100%
1 (Normal)	6	100%	100%	100%

*PPV—positive predictive value.*

**Table 4:** Biological functions of final CRC gene signatures.

Gene Name	Biological Function
Carbonic anhydrase 2 (CA2)	Carbonic anhydrases are a large family of zinc metalloenzymes that catalyze the reversible hydration of carbon dioxide
Carbonic anhydrase 7 (CA7)	
Integral membrane protein 2C (ITM2C)	Enables amyloid β binding activity. Involved in negative regulation of neuron projection development and neuron differentiation.

## Data Availability

Publicly available dataset was used analyzed which are available on the TCGA repository site (https://portal.gdc.cancer.gov/ accessed on 2 March 2023) with project name TCGA-COAD and TCGA-READ and GEO site (https://www.ncbi.nlm.nih.gov/geo/ accessed on 2 March 2023). The accession number for the GEO datasets are (a) GSE50760 and (b) GSE142279.
